# A randomised controlled comparison of early post-pyloric versus early gastric feeding to meet nutritional targets in ventilated intensive care patients

**DOI:** 10.1186/cc8181

**Published:** 2009-11-25

**Authors:** Hayden White, Kellie Sosnowski, Khoa Tran, Annelli Reeves, Mark Jones

**Affiliations:** 1 Department of Critical Care, Logan Hospital, University of Queensland, Armstrong Road, Meadowbrook, Brisbane, 4131, Australia; 2 Department of Nutrition Services, Logan Hospital, Armstrong Road, Meadowbrook, Brisbane, 4131, Australia; 3 School of Population Health, University of Queensland, 15 Butterfield Street, Herston, 4006, Australia

## Abstract

**Introduction:**

To compare outcomes from early post-pyloric to gastric feeding in ventilated, critically ill patients in a medical intensive care unit (ICU).

**Methods:**

Prospective randomized study. Ventilated patients were randomly assigned to receive enteral feed via a nasogastric or a post-pyloric tube. Post-pyloric tubes were inserted by the bedside nurse and placement was confirmed radiographically.

**Results:**

A total of 104 patients were enrolled, 54 in the gastric group and 50 in the post-pyloric group. Bedside post-pyloric tube insertion was successful in 80% of patients. Patients who failed post-pyloric insertion were fed via the nasogastric route, but were analysed on an intent-to treat basis. A per protocol analysis was also performed. Baseline characteristics were similar for all except Acute Physiology and Chronic Health Evaluation II (APACHE II) score, which was higher in the post-pyloric group. There was no difference in length of stay or ventilator days. The gastric group was quicker to initiate feed 4.3 hours (2.9 - 6.5 hours) as compared to post-pyloric group 6.6 hours (4.5 - 13.0 hours) (*P *= 0.0002). The time to reach target feeds from admission was also faster in gastric group: 8.7 hours (7.6 - 13.0 hours) compared to 12.3 hours (8.9 - 17.5 hours). The average daily energy and protein deficit were lower in gastric group 73 Kcal (2 - 288 Kcal) and 3.5 g (0 - 15 g) compared to 167 Kcal (70 - 411 Kcal) and 6.5 g (2.8 - 17.3 g) respectively but was only statistically significant for the average energy deficit (*P* = 0.035). This difference disappeared in the per protocol analysis. Complication rates were similar.

**Conclusions:**

Early post-pyloric feeding offers no advantage over early gastric feeding in terms of overall nutrition received and complications

**Trial Registration:**

Clinical Trial: anzctr.org.au:ACTRN12606000367549

## Introduction

Adequate nutritional support plays a significant role in the outcome of critically ill patients [1]. Furthermore, it is generally accepted that enteral feeding is preferable to parenteral feeding [2-4]. Benefits of enteral feeding may include improvements in intestinal structure and function, prevention of bacterial translocation and infective complications with lower morbidity and cost [5-7]. There is also evidence that early enteral feeding is beneficial and is recommended in a number of guidelines [1,3,8].

Delivery of nutrition via the nasogastric route may be problematic because a significant percentage of intensive care unit (ICU) patients suffer from bowel motility disorders leading to high gastric residual volumes (GRV) and under nutrition [9]. Furthermore, there is a concern that gastric feeding may lead to pulmonary aspiration [10,11]. One potential solution to this problem is the insertion of transpyloric feeding tubes. Small bowel (SB) feeding has the theoretical benefit of improved nutrition and lower aspiration and pneumonia rates [8,10]. However, complications such as misplacement of the small bore feeding tube into the lung with resultant pneumothorax are not rare [12-14]. In addition, insertion of post-pyloric tubes can be time-consuming and costly leading to delays in the initiation of feeds.

A number of post-pyloric insertion techniques have been described. The use of endoscopy or fluoroscopy has a relatively high success rate but is limited by availability, cost and often the need to transfer patients out of the ICU [15-19]. Furthermore, regional ICUs may not have ready access to endoscopy. In fact, Heyland and colleagues recommended post-pyloric feeding as a routine only in those institutions where it could be conveniently and hastily established [20]. Several bedside techniques have been trialled with varying success [21-23]. Some studies have used dedicated physicians or dieticians to place post-pyloric tubes; however, providing 24-hour service may be unachievable. Training ICU nurses to insert SB tubes can overcome delays in feeding and potentially improve overall patient nutrition.

The aim of our study was to compare early gastric with post-pyloric feeding in ventilated, critically ill patients in a regional ICU. Our primary end-points included: the success rate of nurse initiated insertion of post-pyloric tubes, the time taken to insert the tube, time to reach goal feeds and total nutrition received over ICU stay as a proportion of the calculated ideal. As part of the secondary analysis, we compared complication rates between groups including incidence of ventilator associated pneumonia (VAP), GRV and mortality.

## Materials and methods

A single-site, prospective, randomised, controlled trial was conducted over a period of 12 months to compare gastric with post-pyloric feeding in ventilated, critically ill patients. The study setting was an eight-bed regional ICU. The Princess Alexandra Hospital Human Research Ethics Committee gave approval to conduct the investigation. Each patient or their next of kin provided written informed consent to participate.

All patients over 18 years of age admitted to ICU and expected to require mechanical ventilation for more then 24 hours were considered for inclusion. Exclusion criteria included ischaemic bowel, bowel obstruction, exacerbation of inflammatory bowel disease, acute variceal bleeding and patients deemed to be at high risk for anastomotic leak. Patients remained in the study until enteral feeding was ceased or they were discharged from the ICU.

Ventilated patients were randomly assigned to receive enteral feed via a nasogastric or a post-pyloric tube. Randomisation was achieved using a computer-generated random number sequence and a sealed opaque envelope technique. Patients were managed by a number of physicians, most of who were not involved in the study.

All enteric feeding tubes were placed by the bedside nurse. All participants irrespective of allocated group received a nasogastric tube (size 12, 14 or 16) positioned into the stomach. The post-pyloric group received a Corflo^®^-Ultra Lite unweighted feeding tube (VIASYS MedSystems, Wheeling, IL, USA) - 10 FR 109 cm for patients less than 80 kg, or 10 FR 140 cm for patients greater than 80 kg inserted into either the duodenum or jejunum.

All patients received their feeding tubes as soon as possible. ICU nursing staff were trained by the principle researchers or the ICU clinical facilitator to insert the post-pyloric tubes using a blind insertion technique and were required to pass a competency test [24].

### Insertion of post-pyloric tube

In order to limit bias, all participants received 500 mg erythromycin intravenously prior to insertion of the enteric tubes. The insertion of the post-pyloric tube commenced after at least 30 minutes of drug administration. The patient was placed in a semi-supine position at 30°. The Corflo^®^-Ultra Lite feeding tube was inserted into the stomach to a length of approximately 45 cm. Placement was confirmed by auscultating the stomach while insufflating air. As per the manufacturer's instructions, the tube was flushed with 2 ml water to lubricate the guide wire. The tube was advanced very slowly until a further 15 cm of tube had been inserted. The frequent insertion of 5 to 10 ml air checked for kinking or misplacement. The tube continued to be advanced until it reached 90 cm at the nare for a patient under 80 kg, or 110 cm for a patient weighing more than 80 kg. Abdominal radiography confirmed correct positioning. If the tube was not successfully placed after three attempts, further attempts were abandoned, and the patient was fed via the nasogastric route.

An enteral feeding algorithm which prescribed the appropriate type of feed and target rate was developed by the dietetic department. Height was used to estimate the ideal body weight or adjusted body weight for obese patients. The algorithm prescribed 30 kcal/kg of ideal body weight as this was comparable with 1.3 times the resting energy expenditure as determined by indirect calorimetry [25]. The protein requirement selected for the algorithm was 1.5 g/kg ideal body weight, except in the case of liver and renal failure where the requirement was provided at 1 to 1.2 g/kg [26,27].

Enteral feeds were commenced at 40 ml/hour. The nasogastric tube was aspirated every four hours. If the gastric residual was less than 200 ml after four hours, the rate was increased to the recommended target rate. Nasogastric aspirates of greater than 200 ml warranted the use of prokinetic agents. Initially, metoclopramide 10 mg every six hour was prescribed. Erythromycin 250 mg twice daily was added if large aspirates persisted. If GRV remained above 200 ml in the gastric group, a post-pyloric feeding tube was inserted and the feeding regime recommenced. Despite these measures, some participants were unable to successfully absorb enteral feed. These patients progressed to parenteral nutrition.

Once enrolled in the study, demographic data was collected including age, sex and primary diagnosis. An Acute Physiologic and Chronic Health Evaluation (APACHE II) score was determined. Procedural detail included the total time taken to successfully place the post-pyloric tube and the number of attempts required. Times were documented at the commencement of enteral feed and the achievement of the target rate of feed. Other variables recorded included the amount of gastric residual at four-hourly intervals. All episodes of use of prokinetic medication (metoclopramide and erythromycin) were recorded.

Patient outcome data including length of ICU stay and the total number of ventilated and enteral feeding hours was documented. Diagnosis of VAP was based on: new onset (after 48 hours) of fever, leukocytosis, new pulmonary infiltrates on chest radiograph, increased pulmonary secretions and a clinical pulmonary infection score above six [28,29].

Analysis was performed using Stata/IC, version 10 for Windows (StataCorp LP, College Station, TX, USA). Continuous variables are reported as medians and inter-quartile range and compared using a Wilcoxon rank-sum test. A multiple regression model was used to compare the continuous outcomes of the study with adjustment for differences in baseline variables. For positively skewed outcome data, a log transformation was used to remove skew prior to regression analysis. Nutritional outcomes collected on a daily basis were averaged over the follow-up period for individual patients prior to analysis. Fisher's exact test was used to compare binary variables and logistic regression was used to compare mortality rates in the two treatment groups. The primary analysis was by intention-to-treat, however, a secondary per-protocol analysis was also performed. A sample size of 50 patients in each treatment group was pre-specified to have 90% power to detect a halving of the time to reaching goal feeds. This calculation assumed a level of significance of 5% and a modest lost to follow up rate of 10%. Patients were randomly assigned to the two treatment groups using a computer-generated random list.

## Results

A total of 108 patients were randomised, 57 in the gastric group and 51 in the post-pyloric group (Table 1). Of the 108 participants randomised, four were randomised in error and were excluded and two required total parenteral nutrition (TPN) support. Data from 104 patients was analysed. There were 10 patients in the post-pyloric group who did not receive a post-pyloric tube and four patients in the gastric group that did receive a post-pyloric tube. Of the patients in the post-pyloric group who did not receive a post-pyloric tube, 10 were attempted without success, giving an overall success rate of 80%. APACHE II scores for the successful and unsuccessful groups were similar. However, there was a significant difference between the GRV of the group where a post-pyloric tube could not be passed, ie 297 (232 to 442) ml/day compared with 126 (42 to 284) ml/day in the successful group (*P *= 0.027).

**Table 1:** Patient characteristics (intent-to-treat analysis)

Variable	Gastric group Median (IQR)	Post-pyloric group Median (IQR)
Age	54 (40-63)	50 (45-70)
Sex (M:F)	28:26	24:26
APACHE II score	24.5 (20-28)	30 (25-35)^a^
Length of stay in days	5.02 (1.98-9.99)	5.3 (2.73-9.89)
Ventilator days	3.92 (1.5-8.54)	3.93 (2.3-8.38)
Number of interal feed days	3.92 (1.05-7.88)	3.63 (1.89-6.92)
Diagnoses: n (%)		
*Medical*		
Sepsis	7 (13)	9 (18)
Cardiac arrest	9 (17)	5 (10)
Respiratory infection	6 (11)	13 (26)
COAD	4 (7)	4 (8)
Other	21 (39)	16 (32)
*Surgical*		
Trauma	6 (11)	2 (4)
Other	1 (2)	1 (2)

APACHE = Acute Physiologic and Chronic Health Evaluation; COAD = Chronic Obstructive Airways Disease; F = female; IQR = interquartile range; M = male.

^a ^*P *= 0.005.

On intent-to-treat analysis, there was no difference in the length of stay or ventilator days between groups (Table 2). In terms of the primary outcomes the gastric group was quicker to initiate feed as compared with post-pyloric group. The time to reach target feeds from admission was also faster in the gastric group although time to goal from initiation of feeds was similar suggesting the difference is related to the procedure itself rather than feeding intolerance. The average daily energy deficit was lower in the gastric group. There was evidence that a delay in initiation of feeding contributed to this result because the deficits on day one were higher in the post-pyloric group but not significantly so.

**Table 2:** Nutritional data (intent-to-treat analysis)

Variable	Gastric group Median (IQR) n = 54	Post-pyloric group Median (IQR) n = 50	*P *value
Goal feed rate (ml/hour)	74 (69-81)	71.5 (59-79)	0.13
Time to initiate feed from admission or ventilation in hours	4.3 (2.9-6.5)	6.6 (4.5-13.0)	0.0002^a^
Time to reach goal from initiation of feeds in hours	4.3 (4.0-5.0)	4.1 (3.4-5.0)	0.3
Time to reach goal from admission or ventilation in hours	8.7 (7.6-13.0)	12.3 (8.9-17.5)	0.004^b^
Average daily energy required in Kcal	1588 (913-1892)	1463 (1232-1804)	0.7
Average daily energy deficit in Kcal	73 (2-288)	167 (70-411)	0.035
Day 1 energy deficit in Kcal	8 (0-178)	48 (0-361)	0.5
Average daily protein required in grams	69 (45-87)	63 (50-78)	0.5
Average daily protein deficit in grams	3.5 (0-15)	6.5 (2.8-17.3)	0.11
Day 1 protein deficit in grams	1 (0-4)	3 (0-13)	0.4

^a^A comparison adjusting for age and Acute Physiologic and Chronic Health Evaluation (APACHE) II score was also significant (*P *< 0.001), ^b^A comparison adjusting for age and APACHE II score was also significant (*P *= 0.011).

IQR = interquartile range.

Baseline characteristics demonstrated that the post-pyloric group were sicker compared with the gastric group (Table 1). The APACHE II scores were higher and there was an increased incidence of diabetes three (6%) compared with six (12%), acute renal failure nine (17%) compared with 12 (24%) and vasopressor use 20 (37%) compared with 27 (54%) in the post-pyloric group. Higher APACHE II scores were associated with higher energy and protein deficits. Adjusting for these discrepancies led to a non-significant difference in energy deficit between the two groups (Table 3).

**Table 3:** Multiple regression analysis of average daily energy deficit

Variable	Estimate^a^	95% confidence interval	*P *value
Post-pyloric group^b^	1.19	0.96-1.48	0.094
Age	1.00	0.99-1.01	0.9
APACHE II score	1.02	1.01-1.03	0.003

APACHE = Acute Physiologic and Chronic Health Evaluation.

^a^Estimate based on a ratio where 1.0 indicates no difference, ^b^Compared with gastric group.

Complication rates were similar between groups. Average daily GRV were similar although there was a trend toward higher residuals in the post-pyloric group: 190 (55 to 301) ml compared with 111 (43 to 275)ml (*P *= 0.3) in the gastric group. There was also evidence that higher APACHE II score was associated with increased daily average nasogastric aspirate (by 3% for each unit increase in APACHE II score). A comparison of average daily GRV by position of tube (duodenum versus jejunum) showed no difference (*P *= 0.96). In terms of VAP, there were 16 events in total, 5 in the post-pyloric group and 11 in the gastric group (*P *= 0.18).

Drugs that affect gastric motility were recorded. Number of days on fentanyl, morphine, metaclopramide or erythromycin was similar in the two groups: 2 (0 to 5.5) days in the gastric group; 2 (0 to 4) days in post-pyloric group (*P *= 0.7). Number of days on either metaclopramide or erythromycin was similar in the two groups: 0 (0 to 4.5) days in the gastric group; 0 (0 to 2) days in the post-pyloric group (*P *= 0.6). The number of deaths were 5 in the gastric group versus 11 in the pyloric group giving an odds ratio of 2.86 (95% confidence interval (CI) = 0.92 to 8.89, *P *= 0.069). When adjusted for apache score the odds ratio is 2.15 (95% CI = 0.65 to 7.07, *P *= 0.20).

A per-protocol analysis was performed in order to correct for failure of insertion of post-pyloric tube (Tables 4 and 5). Patients who actually received a post-pyloric tube (n = 44) were compared with patients who did not (n = 60). Nutritional differences demonstrated during the intent-to-treat analysis disappeared when examined on a per-protocol basis although gastric feeding still showed a trend toward higher average nutritional intake.

**Table 4:** Patient characteristics (per protocol analysis)

Variable	No post-pyloric tube Median (IQR)	Post-pyloric tube Median (IQR)
Age	53 (42-64)	56 (40-67)
Sex (M:F)	35:29	19:25
APACHE II score	26 (21-31.5)	28.5 (22.5-33.5)
Length of stay in days	4.97 (2.0-10.0)	5.57 (2.8-9.8)
Ventilator days	3.43 (1.6-8.4)	4.92 (2.3-8.2)
Number of interal feed days	3.1 (1.3-6.6)	4.02 (1.9-7.5)

APACHE= Acute Physiologic and Chronic Health Evaluation; F = female; IQR = inter-quartile range; M = male.

**Table 5:** Nutritional data (per protocol analysis)

Variable	No post-pyloric tube Median (IQR) n = 60	Post-pyloric tube Median (IQR) n = 44	*P *value
Goal feed rate (ml/hour)	74 (66-79)	71.5 (62-81)	0.6
Time to initiate feed from admission or ventilation in hours	5.0 (3.6-9.4)	5.8 (4.1-10.0)	0.25^a^
Time to reach goal from initiation of feeds in hours	4.1 (3.8-5.4)	4.1 (3.6-5.3)	0.9
Time to reach goal from admission or ventilation in hours	9.5 (7.6-16.3)	10.5 (8.4-16.1)	0.21^b^
Average daily energy deficit in Kcal	79 (2-340)	149 (74-369)	0.11^c^
Average daily protein deficit in grams	4.3 (0-16.3)	6.6 (2.9-14.8)	0.23^d^

APACHE = Acute Physiologic and Chronic Health Evaluation; IQR = inter-quartile range.

^a^*P *= 0.19;^b^0.57;^c^0.10; ^d^0.15 (after adjustment for age and APACHE II score).

## Discussion

The effectiveness of post-pyloric as compared with gastric feeding has been examined in a number of studies. In general, the results have largely been equivocal. Both Ho and colleagues and Marik and colleagues confirmed this in two meta-analyses [30,31]. Individual studies have produced varying results. Montejo and colleagues found calorie intake to be similar between the nasogastric and nasojejunal group [11]. On the other hand, Kearns and colleagues found the post-pyloric route supplied more calories while Neumann and colleagues found the opposite [32,33]. With the high incidence of gastroparesis present in ICU patients (up to 50%) it seems counterintuitive that gastric feeding can be as successful if not more than post-pyloric feeding [34,35]. Several reasons have been proposed to explain this observation including the longer time taken to insertion of post-pyloric tubes and therefore, later onset of feeding, the potential for more frequent tube related complications in patients fed via the SB (occlusion, dislodgment, accidental withdrawal) and tube position (i.e. duodenal versus jejunal).

We found that patients fed via a nasogastric tube had a shorter time to initiation of feed and time to reach goal feed and lower average daily energy deficit as compared with the post-pyloric group. Although statistically significant, the difference in energy deficit in absolute terms was only 6%, which may not be clinically significant. Our findings compared favourably to other studies where percentage of daily nutritional targets delivered varied from 56 to 80% and time to achieve full nutritional targets varied from 23 to 43 hours in the post-pyloric group [11,32,33,36-38]. This was despite the fact that the APACHE II scores in our study were higher than in previous studies.

There are several possible explanations for the observed differences. Firstly, in 20% of cases, post-pyloric tube insertion was unsuccessful and these patients were fed via the nasogastric route (see below). As analysis was by intent to treat, they were analysed in the post-pyloric group. To adjust for these cases, we performed a protocol-based analysis, which found the groups to be equivalent (although the trend favoured the gastric fed patients). Secondly, there was a delay in reaching goal feeds in the post-pyloric compared with the gastric group, which although not statistically significant may have influenced total nutritional intake. Thirdly, previous studies have noted a greater incidence of tube displacement in post-pyloric fed patients leading to frequent interruption of feeding.

Lastly, the effect of severity of illness on the gastrointestinal system is variable [34,39]. Although we know of no prospective studies linking severity of illness to the ability to absorb feeds, Nguyen and colleagues found that APACHE II score was associated with delayed gastric emptying [39]. The APACHE II scores of our patients were higher then most prior studies suggesting that they were sicker. We also found a significant difference in APACHE II scores between the groups (Table 1). Once results were adjusted for APACHE II score, the difference in nutritional outcomes was not apparent.

A principle motivation for the use of SB feeding is the high incidence of gastroparesis in ICU populations (50% ventilated and 80% head injured) [35,40]. There are numerous potential reasons for this including abdominal surgery, haemodynamic instability, burns, electrolyte abnormalities, fluid overload and the use of vasoactive drugs or analgo-sedation and it is thought that by bypassing the stomach, feeding tolerance can be improved [41,42]. Most ICU feeding studies use GRV as a surrogate for gastric emptying and motility. The utility and significance of this measurement is controversial and depends on a number of factors. Indeed, the relation between GRV and gastric emptying is weak [43,44]. The level of aspirate considered excessive is largely arbitrary and can vary between 150 and 400 ml [45]. Furthermore, up to 25% of patients with GRV more than 150 ml have a normal gastric emptying and can continue on prokinetics.

Neither Ho and colleagues nor Marik and colleagues reported on GRV in their analyses [30,31]. Many individual studies report the number of episodes of high GRV rather than the absolute amount. Montejo and colleagues found that up to 50% of patients had high GRV in the gastric group compared with 2% in the post-pyloric group [11]. Neumann and colleagues, however, found similar results between groups [33]. Differences in definitions of high GRV limit comparison between studies. Montejo and colleagues considered a GRV of 300 ml significant whereas, Neumann and colleagues used 200 ml as a cut off. A high incidence of GRV is considered important because it may increase the risk of aspiration and VAP. In our study we reported on average GRV and found no significant difference, although the GRV in the post-pyloric group was higher. This may partially be explained by the higher APACHE II score and therefore, increased likelihood of gastroparesis in that group. Furthermore, some tubes in the post-pyloric group were in the duodenum rather then jejunum. However, when stratified by tube position, there was no difference in GRV. These findings were in keeping with Heyland and colleagues who similarly failed to find a relation between SB tube position and amount of reflux [10]. Dysmotility in critically ill patients is known to affect the small bowel, and may lead to significant reflux.

Although not statistically significant the mortality rate was higher in the post-pyloric group. The reasons for this are not obvious. As noted, the APACHE II scores were higher in post-pyloric group but even correcting for this, the odds ratio for death was 2.15. All deaths were reviewed and reported to the ethics review committee. No evidence could be found to link the insertion of post-pyloric tubes to the deaths. Furthermore, the mortality rate of 20% in the post-pyloric group was not unexpected given their high APACHE II scores. We therefore do not believe there is any evidence that the insertion of post-pyloric tubes contributed to the deaths of any patient.

Other major complications from post-pyloric tube insertion were rare and we had no significant episodes. We examined the incidence of VAP because there is some evidence that SB feeding may be beneficial. Although this seems a reasonable assumption, there is little strong evidence from the literature to support it [10,30,32,46]. We found a similar incidence of VAP between the groups although there was a trend toward a lower incidence in the post-pyloric group. Our numbers were insufficient to reach statistical significance. The diagnosis of VAP is controversial and diagnostic criteria vary between studies. We did not examine the incidence of aspiration.

Endoscopic guided post-pyloric tube insertion is costly, is not universally available and can lead to significant delays in initiation of feeds. In fact, delays of more then 24 hours are the rule [11,33,47]. The mean time to reach goal feed in the studies reviewed by Ho and colleagues ranged between 23 and 43 hours [30]. We demonstrated that by employing a nurse-initiated bedside insertion method and an aggressive approach to enteral feeding, the time to reach goal feeds can be reduced, as compared with other studies. Our success rate of 80% compares well with other techniques although some have reported higher insertion rates [24,48,49]. Other non-invasive methods have similar outcomes [50-53]. In most instances, the post-pyloric tube was inserted on the first attempt. Despite reports of a high incidence of malposition, there were no episodes of lung insertion.

This study has several limitations. Firstly, doctors were not blinded to treatment group. Secondly, nurses with varying experience were responsible for insertion of post-pyloric tubes. This was intentionally done to reflect the day to day practice of the ICU. Thirdly, there was a 20% failure for insertion of post-pyloric tubes. The blind insertion technique was chosen because access to endoscopic insertion is limited and would have significantly delayed initiation of feeds. This reflects our current practice in the ICU. Fourthly, difference in APACHE II scores suggest the post-pyloric group contained sicker patients, which may have influenced feeding and outcome. And finally, our patient population is largely medical and therefore, may not represent findings in a surgical group of patients.

## Conclusions

Our data largely support the findings of previous meta-analyses, that early post-pyloric feeding is not superior to gastric feeding in the medical ICU population. Whether post-pyloric feeding can be effective in selected patients such as those unable to tolerate gastric feeding is unclear. We also demonstrated that bedside nursing staff can successfully and safely place post-pyloric tubes in the majority of patients, potentially reducing delays in the initiation of feeds.

## Key messages

• Early post-pyloric feeding is no more effective than early gastric feeding.

• Blind insertion of naso-jejunal feeding tubes by bedside nursing staff is highly effective.

## Abbreviations

APACHE II: Acute Physiology and Chronic Health Evaluation II; CI: confidence interval; GRV: gastric residual volumes; ICU: intensive care unit; SB: small bowel; TPN: total parenteral nutrition; VAP: ventilator associated pneumonia.

## Competing interests

The authors declare that they have no competing interests.

## Authors' contributions

HW and KS conceived, managed study and drafted manuscript. AR supplied nutritional data and contributed to manuscript. KT participated in its design and coordination and helped to draft the manuscript. MJ participated in the design of the study and performed the statistical analysis. All authors read and approved the final manuscript.
